# Monitoring of frying process in canola oil blend using fourier transform infrared and chemometrics techniques

**DOI:** 10.1002/fsn3.2558

**Published:** 2021-09-04

**Authors:** Muhammad Haseeb Ahmad, Zainab Shahbaz, Muhammad Imran, Muhammad Kamran Khan, Niaz Muhammad, Sanaullah Iqbal, Waqas Ahmed, Tanvir Ahmad

**Affiliations:** ^1^ Department of Food Science Faculty of Life Sciences Government College University Faisalabad Pakistan; ^2^ National Agriculture Education College Kabul Afghanistan; ^3^ Department of Food Science and Human Nutrition Faculty of Bio‐Sciences University of Veterinary & Animal Sciences Lahore Pakistan; ^4^ Department of Statistics Faculty of Physical Sciences Government College University Faisalabad Pakistan

**Keywords:** canola, D‐optimal design, fourier transform infrared, partial least square regression, principal components analysis

## Abstract

The production of trans‐fats and chemical changes during the process of frying are serious public health concerns and must be monitored efficiently. For this purpose, the canola oil was formulated with different ratio of extra virgin olive oil and palm olein using D‐optimal mixture design, and the best formulation (67:22:11) based on free fatty acid (FFA) content, peroxide value (PV), and iodine value (IV) as responses was selected for multiple frying process. The data on FFA, PV, and IV along with Fourier transform‐infrared (FT‐IR) spectra were taken after each frying up to ten frying. The spectral data were preprocessed with standard normal variate followed by principal component analysis which is clearly showing the differentiation for various frying. Similarly, partial least square regression was applied to predict the FFA (0.37%–1.63%), PV (4.47–13.85 meqO_2_/kg), and IV (111.51–51.39 I_2_/100 g) which demonstrated high coefficient of determination (R^2^) 0.84, 0.83, and 0.81, respectively. It can be summarized that FT‐IR can be used as a novel tool for fast and noninvasive quality determination of frying oils.

## Graphical abstract

The current study was designed to make canola oil blends with different concentrations of olive oil and palm olein. These blends were optimized by D‐optimal Design using the oil stability parameters such as free fatty acid (FFA), peroxide (PV), and iodine values (IV). The optimized canola oil blend was further used for multiple frying, and FT‐IR spectral fingerprints were taken which is considered to be a rapid, non‐destructive, and environment‐friendly analytical tool for characterization of the chemical changes taken place due to the processing.

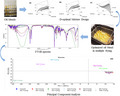

## 1 INTRODUCTION

Fats and oils play a crucial role in our daily food consumption as they give us vital fatty acids and intense energy supply. Frying in fats and oils is one of very old techniques used in processing of food for quick preparation and provides some special effect on sensation of food (Bou et al., 2012). This deep fat frying (DFF) process is performed by fully immersing the foods in hot oil based on the continuous heat and the mass transfer at elevated temperatures ranging from 150 to 200°C (Moreira, 2014). DFF efficiency relies on either the process of frying itself, the nature of the food, and/or type of frying oil (Marikkar et al., 2011). During the process, thermo‐oxidation and cleavage of triglycerides occur at higher temperatures in the presence of oxygen, particularly if the oil is polyunsaturated, resultantly, the formation of volatile and nonvolatile oxidative compounds. Repeated and persistent use of fried oils produces undesirable compounds that may affect nutritional quality and pose potential threats to the nutrition and human health (Andrikopoulos et al., 2002). Pure oils present low stability against oxidation during frying, alternatively, encouraging the use of mixture of oils for better stability (Alireza et al., 2010). Mixing two or more oils with distinct properties is among the techniques for developing new special oil products. In addition to improving the fatty acids' profile, combining multiple forms of vegetable oils often increases the amounts of natural antioxidants and bioactive phytonutrients in the blends and offers higher quality stable oils (Tiwari et al., 2014). Therefore, first a blend of oil having canola, extra virgin olive oil, and palm olein was optimized using D‐optimal design against different quality indicators of frying oils.

Currently, the legislative authorities and food industries are looking for analytical solutions for rapid and noninvasive quality control tools. Different spectroscopic techniques such as Fourier Transform Infrared (FT‐IR) have now been extensively studied to analyze various chemical changes and correspond such changes with instrumental data using statistical tools importantly chemometrics. FT‐IR is an analytical instrument based on the scanning of samples using infrared light to identify polymeric, chemical, and inorganic materials (Velasco & Dobarganes, 2002). The sample detects some of the infrared radiation and produces its molecular fingerprint in the form of a spectrum. The difference in the characteristic pattern of the absorption bands indicates a modification in the substrate composition or existence of contaminants (Chippie et al., 2002). Furthermore, the sensitivity and precision of FT‐IR detectors and a wide variety of automated algorithms have greatly increased the realistic use of infrared for quantitative research analysis. This apparatus is quick to establish and calibrate quantitative approaches and can be used in basic repetitive analytical procedures (Ma, 2018).

Different studies have been conducted on the application of FT‐IR to analyze the quality of either adulterated oil, frying oil, or fried products, for instance, monitoring frying quality of palm and soybean oils used for preparing Falafel (Al‐Degs et al., 2011), determination of peroxide value of red fruit oil (Andina et al., 2017), degree of degradation of frying rapeseed oil (Chen et al., 2015), monitoring of free fatty acids in commercially available *Nigella sativa* oil (Mahesar et al., 2014), tracking performance of virgin coconut oil during frying (Srivastava & Semwal, 2015), iodine value of blends with refined soybean oil, peanut oil, rapeseed oil and sunflower seed oil (Meng et al., 2017), correlating the acid values with FT‐IR spectroscopy (Jiang et al., 2016), adulteration in wheat germ oil (Arslan & Çağlar, 2019), and others. All these monitoring studies based on spectroscopic techniques are generally combined with suitable chemometrics statistical models (Amit et al., 2020). Unfortunately, no data were found on the application of FT‐IR on oil blends which are commercially available and are used for frying at consumer level.

Based on the literature cited above, the present study was planned to optimize the suitable blend for frying using D‐optimal design statistical tool and then observe the chemical changes during frying by FT‐IR. These chemical changes observed in the form FT‐IR spectra were correlated with hectic and time‐consuming conventional analytical methods with help of chemometrics.

## 2 MATERIALS AND METHODS

### 2.1 Raw material

Canola oil, extra virgin olive oil (EVOO), and frozen French fries were purchased from market of Faisalabad‐Pakistan. Palm olein (degummed and neutralized) was procured from the local oil processing industry located at Faisalabad, Pakistan.

### 2.2 D‐optimal mixture design for oil blends

D‐optimal mixture design was applied to develop formulations of canola oil with EVOO and palm olein in different ratios with the final volume of each formulation equal to 100 (table 1). Generally, D‐optimal mixture design was applied as an efficient statistical tool to determine the best combination among wide range of studied components (Mohamad Zen et al., 2015). Best suggested formulation of oil blend was interpreted from the chemical analyses as response variables and was further subjected to multiple frying with structural description using FT‐IR spectroscopy. The quadratic regression model with three factors is as follows:

$$R=\alpha_{0}+\sum_{n=1}^{\infty }\alpha_{n}F_{n}+\sum_{n=1}^{\infty }a_{n}^{2}F_{n}^{2}+\sum_{n<m}^{\infty }\alpha_{\mathrm{nm}}F_{n}F_{m}.$$

**TABLE 1:** D‐optimal design suggested formulations and chemical analyses of canola oil blends after frying

Formulation	Composition			Chemical Analyses		
	Canola oil (% v/v)	EVO oil (% v/v)	Palm olein (% v/v)	Free fatty acid (%)	Peroxide value (meq O_2_/kg of oil)	Iodine value (g of I_2_/100 g oil)
*F_1_*	55	30	15	0.49	6.2	94.57
*F_2_*	55	30	15	0.47	6.4	96.13
*F_3_*	73	22	5	0.63	9.6	86.87
*F_4_*	67	22	11	0.42	4.9	109.05
*F_5_*	67	28	5	0.44	5.1	107.05
*F_6_*	59	27	14	0.48	5.3	103.47
*F_7_*	75	15	10	0.62	9.2	87.35
*F_8_*	70	15	15	0.60	8.2	89.76
*F_9_*	61	30	9	0.44	5.4	101.35
*F_10_*	80	15	5	0.66	10.1	84.05
*F_11_*	80	15	5	0.67	10.3	83.05
*F_12_*	66	19	15	0.47	6.2	98.24
*F_13_*	62	23	15	0.46	5.3	93.08
*F_14_*	70	15	15	0.59	8.0	91.76

### 2.3 The process of frying

The process of frying was performed in a “Cool Touch Electric Deep Fryer” (Anex, AG‐2012). Fourteen formulations with different combinations of canola oil, EVOO, and palm olein blends were used in frying as per D‐optimal design. For each trial, 50 g of frozen French fries was fried in 1 L of preheated oil blend at a temperature of 180°C for 4 min. After each frying process, oil samples were collected, cooled at ambient temperature, and subjected to chemical analysis. After screening out the best formulation using D‐optimal mixture design, the safety and quality of oil blend were tested up to 10 frying using the above described procedure.

### 2.4 Chemical analysis

#### 2.4.1 Free fatty acid

Free fatty acid (FFA) was found out by adopting the AOCS Official Methods Ca 5a‐40 (Firestone, 2009). For this purpose, a 10 g of canola oil blend was taken into a conical flask (250 ml) and heated with 100 ml ethanol with addition of 1 ml of phenolphthalein solution as indicator. The mixture was then boiled for about 4–5 min and titrated against 0.1 N KOH till the persistence of pink color for at least 30 s. The value of FFA was calculated using the following formula:

(1) $$\text{FFA =}\;\frac{56.1\times V\times N}{W}.$$

where 56.1 molecular weight of potassium hydroxide, V = Volume of potassium hydroxide used, N = Normality of potassium hydroxide solution, W = Weight of the sample

#### 2.4.2 Peroxide value

Peroxide value is defined as milli‐equivalents of oxygen in one kilogram of oil (meq O_2_/kg of oil) and was determined by following the AOCS Official Methods Ca 5a‐40 (Firestone, 2009). Approximately, 5 g of canola oil blend was taken in conical flask (250 ml) fitted with a plastic stopper and stirred with glacial acetic acid (30 ml), chloroform (20 ml), and potassium iodide solution (0.5 ml). A change in color was appeared. The mixture was further diluted with 30 ml of distilled water and left for a minute while swirling the flask occasionally. Few drops of starch were added to mixture as indicator which turned the solution to dark blue color. The mixture was titrated against 0.01 M of sodium thiosulfate solution till the dark blue color turned into a colorless solution. A blank test was run without the oil sample, and PV was calculated using the following formula:

(2) $$\text{PV =}\;\frac{(V_{s}‐V_{b})N\times 1000}{W}.$$

where Vs = Volume of sodium thiosulfate titrated, Vb = Volume of sodium thiosulfate used in a blank test, N = Normality of sodium thiosulfate solution, W = Weight of sample.

#### 2.4.3 Iodine value

The AOCS Official Methods Ca 5a‐40 (Firestone, 2009) was followed to determine the iodine value. For this purpose, a 0.25 g of canola oil blend was taken into conical flask (500 ml) with a plastic stopper and combined with 25 ml of each CCl_4_ and Wij's solution. The mixture was placed in the dark for 30 min, and 50 ml of 10% potassium iodide solution was added. After diluting the whole mixture with 100 ml of distilled water, titration was executed against standardized sodium thiosulfate solution (0.1 N) using 0.5% starch as indicator. The titration process was stopped on the disappearance of blue color of starch solution. The same procedure was adopted to conduct blank test and value was calculated as:

(3) $$\text{IV}=\frac{12.69(B‐S)N}{W}.$$

where B = Volume of standard sodium thiosulfate solution used for the blank, S = Volume of standard sodium thiosulfate solution used for the sample, N = Normality of standard sodium thiosulfate solution, W = Weight of sample.

### 2.5 FT‐IR spectroscopy

The fourier transform infrared (FT‐IR) spectroscopy is a useful tool for investigating the chemical modifications in edible oils, both qualitatively and quantitatively, while using little amount of sample and minimizing the error (Xu et al., 2016). Spectroscopic data of oil samples after each frying were obtained with an infrared spectrometer (Alpha II FT‐IR, Bruker) fitted with an attenuated total reflection (ATR) accessory. Each sample was preconditioned in a water bath at 40°C for 10 min to avoid any obscurity in the samples. For spectrum, a little quantity of the oil sample was evenly dropped on the crystal surface of the ATR accessory fitted with horizontal ZnSe crystal. Spectrum of each sample was acquired in transmission mode with the range from 4000 cm^−1^ to 400 cm^−1^. Each spectrum was outcome of 16 scans at a resolution of 2 cm^−1^ with FT‐IR spectrometer which is systematized with the latest OPUS software version 7.0. All spectral data obtained through the OPUS software were then transferred to Unscrambler software (CAMO) for chemometrics.

### 2.6 Statistical and chemometric data analyses

The statistical software package Design Expert 7.0 (Stat‐Ease Inc.) was used to develop the formulations of canola oil blends using D‐optimal design and get optimized and best formulation based on the chemical analyses.

#### 2.6.1 Preprocessing of spectral data

The optimized canola oil blend was used for frying of French fries to observe the structural and chemical changes in frying oil. Optimized canola oil blend was taken in triplicate and used for potatoes frying up to ten times. Three spectral fingerprints were taken after each frying operation, and their average was calculated. Then, the mean spectral signatures were pretreated using various preprocessing techniques. Different types of preprocessing treatments such as Standard normal variate (SNV), multiplicative scatter correction (MSC), normalization, and base line corrections were applied to remove the abnormalities in the data datasets (Lee et al., 2018).

#### 2.6.2 Principal components analysis

Principal components analysis (PCA) is one of the most widely and unsupervised chemometric tool used for exploratory data analysis. It reduces the dimensionality and provides the pattern recognition without loss of information. The SNV preprocessed data were utilized for application of PCA using single value decomposition algorithms and full cross‐validation process (Ahmad et al., 2016).

#### 2.6.3 Partial least square regression

Partial least square regression **(**PLSR) models are being used for determination of various parameters. The preprocess datasets (SNV, MSC, normalization, and baseline correction) were subjected to PLSR modeling using leave one out cross‐validation. In this type of modeling, the calibrated models were developed using ten spectral signatures and the eleventh was left out for the prediction models. In this way, all the samples were left out once for prediction models for determination of FFA, PV, and IV using FT‐IR spectral fingerprints after each frying process. The accuracy of the models was determined using root mean square error of cross‐validation (RMSECV) and coefficient of determination (R^2^). The RMSECV and R^2^ can be calculated by using the Equations 4 and 5, respectively.

(4) $$RMSECV=\sum_{i=1}^{n}{\left(m_{i}‐P_{i}\right)}^{2}/n.$$

(5) $$R^{2}=1‐\sum_{i=1}^{n}{\left(m_{i}‐P_{i}\right)}^{2}/\sum_{i=1}^{n}{\left(m_{i}‐\bar{m}\right)}^{2}.$$

Here, n, mi, pi, and $\bar{m}$ stand for number of samples, measured, predicted, and mean value, respectively.

## 3 RESULTS AND DISCUSSION

### 3.1 Optimization of canola oil blend

**FIGURE 1:**
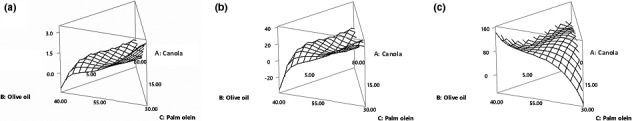
Response surface plots for free fatty acid (a), PV (b), and IV (c)

D‐optimal statistical design was used to formulate the 14 combinations randomly with each contributing the total amount equal to 100 (table 1). All the response values of these combinations were in the quality, and safety confines as set by the regulatory authorities. It was also observed that the contribution of different oils had a significant effect on the chemical quality of the frying oil. The values of dependent variables (FFA, POV, and IV) in design were fit to the response surface linear, quadratic, special cubic, and inverse models and have been represented in the response surface plots (Figure 1). The models had shown high reliability as the values of predicted coefficient of determination (*R* ^2^ = 88.77%) and observed coefficient of determination (*R^2^(obs*.*)*=88.73%) was close to each other.

The free fatty acid (FFA) content shows the degree of oil degradation due to hydrolysis reactions of lipids, cleavage, and oxidation of double bonds of unsaturated fatty acids. After overviewing the observed values, it can be seen that formulations of oil blend (F_10_, F_11_) with canola 80%, olive 15%, and palm olein 5% had higher FFA deterioration and lowest deterioration for F_4_ with canola 67%, olive 22%, and palm olein 11%. The inclusion of olive oil in the blend leads to maintain the quality of all the oil blends due to the presence of phenolic fractions (Del Carlo et al., 2004). Canola oil recorded the maximum oleic acid content, thus a reduced risk of coronary heart attack with high levels of monounsaturated fatty acids in oils (Alireza et al., 2010).

The peroxide value (PV) is a commonly used tool for measuring the initial stage of lipid oxidation which represents the accumulation of peroxides and hydroperoxides. The lowest PV was observed in formulation F_4_ (Canola: EVOO: Palm olein = 67:22:11) while higher values were observed for formulation F_10_ and F_11_ (Canola: EVOO: Palm olein = 80:15:5). Farhoosh et al. (2009) concluded that oil blends with more canola oil have greater peroxide content than blends with less canola oil and consequently high linolenic acid content due to higher PV. The PV value of the blends was improved after the addition of palm olein. The higher olive content in the blends indicated a lower PV value. This implies that natural antioxidants reduce the oxidation process in palm olein and olive oil (Kavuncuoglu et al., 2017). However, peroxides are reactive compounds that are instantly damaged at frying temperatures and decomposed as secondary oxidation components. The use of PV as a standard procedure for the measurement of frying oils also has certain limitations.

The iodine value (IV) is defined as a measure of the degree of unsaturation and is widely used to classify fats and oils. Formulation of oil blend (F_4_) with highest IV (Canola: EVOO: Palm olein = 67:22:11) and that of lowest for blends F_10_, F_11_ (Canola: EVOO: Palm olein = 80:15: 5) were observed. Higher iodine values of oil are vulnerable to oxidation and polymerization. Iodine value reduction is an indicator of lipid oxidation and is consistent with a reduction of double bonds as oxidized by oil (Alireza et al., 2010). However, the protective role of antioxidants which were counted due to the presence of EVOO resulted in smaller decrease in the double bond.

The formulation with the minimum desirability for FFA and POV while that of with maximum for IV was selected on the basis of the agreed limits. After processing of the statistical design, the optimum oil blend obtained was consisted of on average 66.67% canola oil, 22.48% extra virgin olive oil, and 10.85% palm olein which were round off to 67:22:11, respectively. This optimization might correlate with suitable fatty acid composition and equal amount of saturated to unsaturated fatty acids (De Leonardis & Macciola, 2012).

### 3.2 Chemical characterization in multiple frying

The optimized canola oil blend (67:22:11) was further subjected to multiple frying and chemically characterized by FFA, PV, IV, and FT‐IR after each frying. During multiple frying, the amount of FFA increased slowly and did not reach the limit of 2% established by European regulations (table 2). Due to thermal and oxidative decomposition of oils at high temperatures, the rise in free fatty acid values of oils can be attributed to the degradation of long carbon chains into shorter carbon chains. During high‐temperature heating of oils which are ultimately oxidized into low molecular mass fatty acids, FFA formation is due to the cleavage and oxidation of double bonds to form carbonyl compounds (Latha, 2016). Similarly, during frying, the chain reaction in oils develops very active peroxides in auto‐oxidative reactions under the influence of light, oxygen, moisture, heavy metal ions, and higher temperatures. So PV is also a valuable screening tool of the early stages of rancidity arising under moderate conditions and the quality of the lipid matrix. Several studies recorded that secondary oxidized oil products are typically toxic for human health (Gotoh & Wada, 2006). Consequently, in order to protect against the oxidation of fat and oil and the development of secondary oxidized products from food stability and food safety standpoint, the formation of hydroperoxide, the main oxidized product of fat and oil, must be suppressed. The degree of unsaturation is correlated with the melting point and oxidative stability, and IV offers an approximation of these consistency factors. A decrease in iodine value is associated with a decrease in the number of double bonds which became oxidized during the frying process (Alireza et al., 2010).

**TABLE 2:** Chemical analysis of optimized oil blend after multiple frying

No. of frying	Free fatty acid (%)	Peroxide value (meq O_2_/kg of oil)	Iodine value (g of I_2_ /100 g oil)
0	0.37 ± 0.003	4.47 ± 0.08	111.51 ± 1.06
1	0.44 ± 0.002	4.51 ± 0.05	109.37 ± 0.75
2	0.52 ± 0.006	4.87 ± 0.07	104.19 ± 1.09
3	0.61 ± 0.005	5.12 ± 0.09	99.02 ± 0.99
4	0.69 ± 0.002	6.37 ± 0.11	93.84 ± 0.86
5	0.76 ± 0.004	7.62 ± 0.10	89.35 ± 0.97
6	0.94 ± 0.003	8.08 ± 0.04	84.07 ± 1.08
7	1.02 ± 0.001	9.67 ± 0.07	79.72 ± 0.79
8	1.28 ± 0.003	10.34 ± 0.09	67.63 ± 0.84
9	1.47 ± 0.002	12.12 ± 0.06	59.11 ± 0.91
10	1.63 ± 0.005	13.85 ± 0.10	51.39 ± 0.88

**FIGURE 2:**
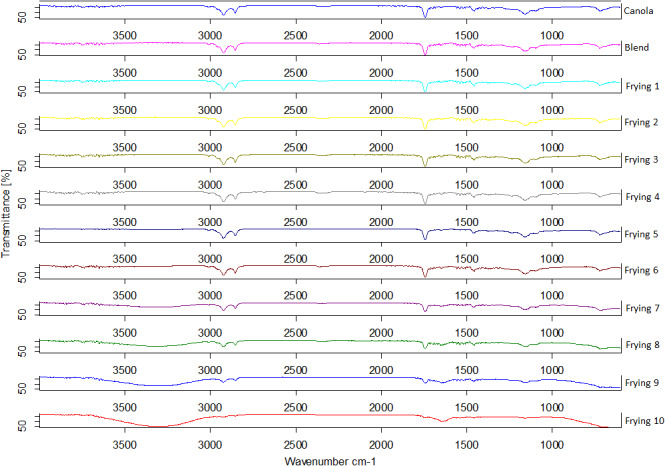
Fourier transform‐infrared spectra for canola oil, optimized blend, and oil samples after each frying

The chemical changes analyzed through FFA, PV, and IV have also been confirmed with FT‐IR spectra during multiple frying (Figure 2). The spectral bands of fried oils obtained were correlated with the available literature. The spectral peaks at 1110 cm^−1^ and 1710 cm^−1^ can be allied to C–O and carbonyl (C = O) bonds of aliphatic esters, respectively (Shen et al., 2014). Next to these peaks, two more strong peaks at 2820 cm^−1^ and 2990 cm^−1^ can be assigned to symmetrical and asymmetrical C–H bonds of CH_2_ structures. With the increase in number of frying, the generic transmittance peaks were diminished and new transmittance peaks were appeared at 1595 cm^−1^ and 3320 cm^−1^ which indicate the formation of saturated bonds (C–O) and FFA formed after the hydrolysis of ester groups. The information obtained through the spectroscopic data is presenting the noticeable differences among various frying; however, it is essential to apply multivariate calibration to validate the information from such complex and overlapping spectra (Jamwal et al., 2021).

### 3.3 Principal component analysis for FT‐IR spectra

**FIGURE 3:**
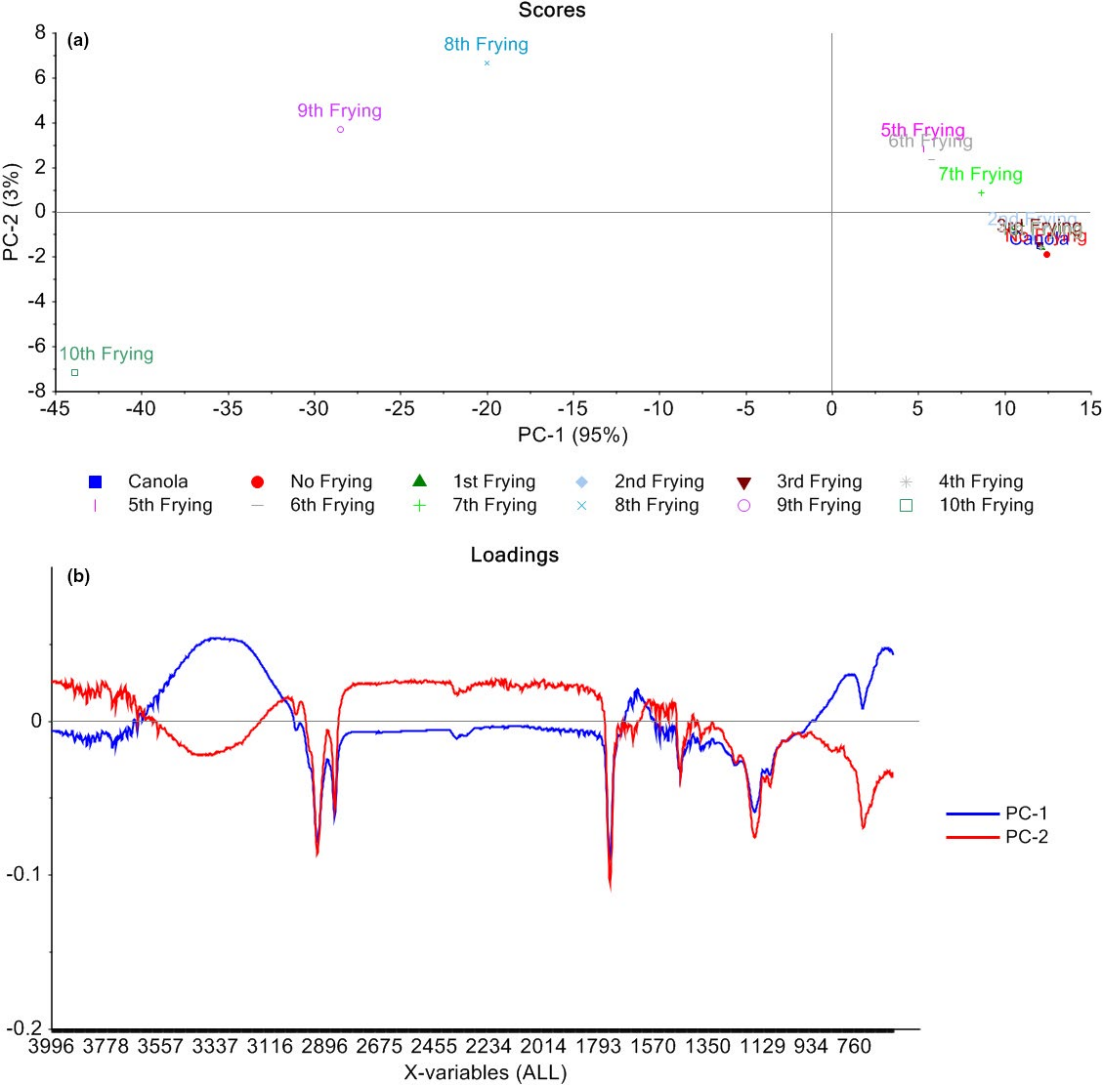
Principal components analysis score plots (a) and SNV preprocessed loadings (b) for multiple frying

PCA was applied on the SNV preprocessed data which shows clear differentiation among different frying of optimized canola oil blend (Figure 3a). The first principal component (PC1) explains 95% variance whereas the second principal component (PC2) provides its share of only 3%. Against PC1, maximum value was observed for canola oil blend with no frying whereas the lowest value was recorded for the oil blend after 10th frying. On the other hand, the maximum scores were recorded oil blend after eighth frying whereas lowest scores were found for oil blend after 10th frying. Hence, only first two PCs provide the maximum variance in the spectral datasets taken after each frying. It can also be observed that up to fifth frying the stability of the oil blend was not affected as one can see the spectral points overlap each other, but after sixth and seventh frying, the changes were observed. However, the most changes were taken place after eighth frying onward as they lie away from each other. These variations in the spectral fingerprints can be observed from the loading plots for PC1 and PC2 as presented in Figure 3b. The loading plot for PC1 shows peaks in various regions of the spectral fingerprints that may responsible for this clear differentiation due to multiple frying of canola oil blend. The peaks were observed in 3600–3200 cm^−1^, 2900–2700 cm^−1^, 1500–1700 cm^−1^, 1200–1000 cm^−1^, and 700–500 cm^−1^ against PC1 and PC2 loadings. These peaks can be correlated to different structural changes taken place due to the frying operation that ultimately results in the differentiation of oil after multiple frying. It can be inferred from above discussion that PCA can differentiate the oil spectra taken after multiple frying.

### 3.4 Effect of preprocessing of FT‐IR spectra and PLSR model

Preprocessing of the spectral fingerprints plays vital role to obtain more robust and accurate models as it removes the noise and other abnormalities lies in the spectral dataset. Different types of preprocessing were applied before development of PLSR model. First of all, the spectral signatures were preprocessed using SNV. After SNV preprocessing, the spectra were used to predict the FFA, PV, and IV using PLSR models with leave one out cross‐validation. The PLSR models developed on SNV preprocessed data showed good prediction as high value of *R* ^2^ for calibration (.84) and *R* ^2^ cross‐validation (.83) was noted along with smaller RMSEC (0.16%) and RMSECV (0.19%) for determination of FFA out of spectral signatures. Similarly, PV of the canola oil blend was also predicted using FT‐IR spectral signatures with SNV preprocessed data. High correlation between spectral signatures and PV was observed as *R* ^2^ at .83 and .82 for calibration and cross‐validation was recorded with 6% RMSEC and RMSECV. The prediction of IV from the spectral signatures shows the similar trend using PLSR model on SNV preprocessed dataset.

Multiplicative scatter calibration (MSC) preprocessing was subjected to spectral fingerprints taken after multiple frying which was further utilized for the development of PLSR model. The improvement in PLSR models was observed as *R* ^2^ for calibration and cross‐validation was found higher as compared to the PLSR model developed on SNV preprocessed data. Here, the *R* ^2^ for calibration .99, .96, and .98 was noted for FFA, PV, and IV, respectively. On the other hand, *R* ^2^ for cross‐validation .87, .86, and .87 was recorded for FFA, PV, and IV, respectively, which show the accuracy and robustness of the fitted models. In addition to this, RMSEC and RMSECV were found less than 5% which are considered to be acceptable for the validation of PLSR models.

Furthermore, area normalization was also applied as preprocessing tool and PLSR model was developed on the spectra taken after multiple frying. It was found that normalization also provided the comparable prediction models with MSC. The *R* ^2^ for calibration and cross‐validation was recorded .98, .99, and .98 for FFA, PV, and IV, respectively, whereas *R* ^2^ cross‐validation was found .84, .76, and .84 for FFA, PV, and IV, respectively. The RMSEC and RMSECV were found less than 5% that is considered to be acceptable for the authentication of PLSR model. It can be observed from the results that preprocessing of spectral signatures is considered to be important as it improves the prediction models. The best PLSR models were built using MSC preprocessing tool. The SNV preprocessing and normalization also show the comparable results as evident from table 3.

**TABLE 3:** PLS models applied on FT‐IR preprocessed data for prediction of oil stability parameters after multiple frying

Preprocessing method	No. of latent variables	Model factors	Free fatty acid	Peroxide value	Iodine value
Standard Normal Variate	1	*R* ^2^_C_	.84	.83	.81
		*R* ^2^_CV_	.83	.82	.79
		RMSEC	.16	1.39	8.57
		RMSECV	.19	1.41	9.82
Multiplicative Scatter Correction	5	*R* ^2^_C_	.99	.96	.98
		*R* ^2^_CV_	.87	.86	.87
		RMSEC	.03	.56	2.38
		RMSECV	.16	.92	7.48
Area Normalization	6	*R* ^2^_C_	.98	.99	.98
		*R* ^2^_CV_	.84	.76	.85
		RMSEC	.04	.28	1.55
		RMSECV	.17	.72	2.78

Note

CV, Cross‐validation; *R* ^2^, Coefficient of determination for calibration; RMSEC, Root mean square error of calibration; RMSECV, Root mean square error of cross‐validation.

## 4 CONCLUSION

The best canola oil blend was formulated with olive oil and palm olein which is usually more stable in frying as compared to pure single oil. Moreover, FT‐IR spectral data in combination with PCA show clear differentiation for the oil samples acquired after multiple frying. Similarly, PLSR model was used to predict FFA, PV, and IV. The changes in chemical analyses and on FT‐IR fingerprints during the process of multiple frying are mainly attributed to the hydrolysis of lipids, breakdown of double bond, and oxidation of oil due to high temperatures and the presence of oxygen. All these changes are important to consider the stability of oil that is ultimately important for the production of good quality fried product having least health concerns. Previously, these changes were governed using chemical‐based standard methods that are laborious and time taking. The prediction of these parameters helps in the development of fast and noninvasive tools for regulatory bodies and industrial applications which may smoothen the way toward sensor development.

## CONFLICTS OF INTEREST

The authors declare that there are no conflicts of interest regarding the publication of this paper.

## AUTHOR CONTRIBUTIONS

**Muhammad Haseeb Ahmad:** Conceptualization (equal); Data curation (equal); Project administration (equal); Software (equal); Writing‐original draft (equal). **Zainab Shahbaz:** Formal analysis (equal); Methodology (equal). **Muhammad Imran:** Methodology (equal). **Muhammad Kamran Khan:** Conceptualization (equal); Project administration (equal); Resources (equal); Supervision (equal); Writing‐review & editing (equal). **Niaz Muhammad:** Validation (equal); Writing‐review & editing (equal). **Sanaullah Iqbal:** Methodology (equal); Resources (equal). **Waqas Ahmed:** Methodology (equal). **Tanvir Ahmad:** Formal analysis (equal); Software (equal).

## Data Availability

All the data that support the findings of this study are included in the manuscript.
